# Supplementary material for: Effectiveness and Safety of Ayurvedic Medicines in Type 2 Diabetes Mellitus Management: A Systematic Review and Meta-Analysis
Source: Front Pharmacol. 2022 Jun 8;13:821810. doi: 10.3389/fphar.2022.821810 (PMC9213670; doi:10.3389/fphar.2022.821810)
Supplement: Supplementary file 1 [file DataSheet1.zip › Supplementary Material/Forest and Funnel Plots/Portulaca oleracea/LDL-C.pdf]

Portulaca oleracea - LDL-C

Study	Intervention N	Intervention Mean	Intervention SD	Comparator N	Comparator Mean	Comparator SD	Mean Diff. with 95% CI	Weight (%)
Farzanegi 2014	14	69.00	8.66	14	82.05	8.10	-13.05 [-19.26, -6.84]	80.39
Dehghan 2016	16	76.35	45.33	16	78.80	50.65	-2.45 [-35.76, 30.86]	2.80
Wainstein 2016	31	112.70	29.50	32	118.50	25.40	-5.80 [-19.38, 7.78]	16.82
**Overall**							-11.53 [-17.10, -5.97]	

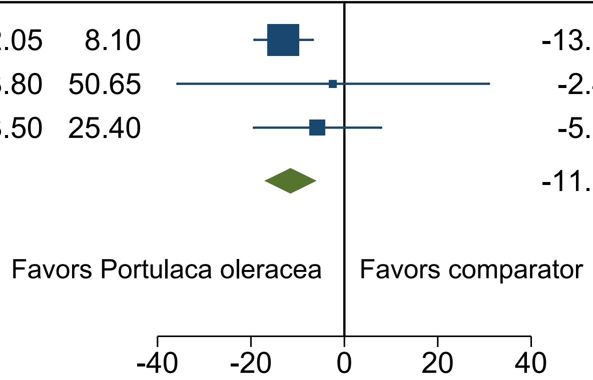

Heterogeneity: $\tau^2 = 0.00$, $I^2 = 0.00\%$, $H^2 = 1.00$

Test of $\theta_i = \theta_j$: $Q(2) = 1.20$, $p = 0.55$

Test of $\theta = 0$: $z = -4.06$, $p = 0.00$

Random-effects REML model
